# Upregulation of miR21 and Repression of Grhl3 by Leptin Mediates Sinusoidal Endothelial Injury in Experimental Nonalcoholic Steatohepatitis

**DOI:** 10.1371/journal.pone.0116780

**Published:** 2015-02-06

**Authors:** Sahar Pourhoseini, Ratanesh Kumar Seth, Suvarthi Das, Diptadip Dattaroy, Maria B. Kadiiska, Guanhua Xie, Gregory A. Michelotti, Mitzi Nagarkatti, Anna Mae Diehl, Saurabh Chatterjee

**Affiliations:** 1 Environmental Health and Disease Laboratory, Department of Environmental Health Sciences, University of South Carolina, Columbia, SC, 29208, United States of America; 2 Free Radical Metabolism Group, Institute of Environmental Health Sciences, Research Triangle Park, NC, 27709, United States of America; 3 Division of Gastroenterology, Duke University, Durham, NC, 27707, United States of America; 4 Dept. of Pathology, Microbiology and Immunology, University of South Carolina School of Medicine, Columbia, SC, 29209, United States of America

## Abstract

Sinusoidal endothelial dysfunction (SED) has been found to be an early event in nonalcoholic steatohepatitis (NASH) progression but the molecular mechanisms underlying its causation remains elusive. We hypothesized that adipokine leptin worsens sinusoidal injury by decreasing functionally active nitric oxide synthase 3 (NOS)3 via miR21. Using rodent models of NASH, and transgenic mice lacking leptin and leptin receptor, results showed that hyperleptinemia caused a 4–5 fold upregulation of hepatic miR21 as assessed by qRTPCR. The upregulation of miR21 led to a time-dependent repression of its target protein Grhl3 levels as shown by western blot analyses. NOS3-p/NOS3 ratio which is controlled by Grhl3 was significantly decreased in NASH models. SED markers ICAM-1, VEGFR-2, and E-selectin as assessed by immunofluorescence microscopy were significantly up regulated in the progressive phases of NASH. Lack of leptin or its receptor in vivo, reversed the upregulation of miR21 and restored the levels of Grhl3 and NOS3-p/NOS3 ratio coupled with decreased SED dysfunction markers. Interestingly, leptin supplementation in mice lacking leptin, significantly enhanced miR21 levels, decreased Grhl3 repression and NOS3 phosphorylation. Leptin supplementation in isolated primary endothelial cells, Kupffer cells and stellate cells showed increased mir21 expression in stellate cells while sinusoidal injury was significantly higher in all cell types. Finally miR21 KO mice showed increased NOS3-p/NOS3 ratio and reversed SED markers in the rodent models of NASH. The experimental results described here show a close association of leptin-induced miR21 in aiding sinusoidal injury in NASH.

## Introduction

With obesity assuming pandemic proportions in the recent decades, incidences of nonalcoholic steatohepatitis (NASH) are on the rise. NASH and its associated comorbidities have a poor prognosis and molecular pathways for causation of this metabolic disease are still emerging [1–3]. Recent hypothesis points towards a multiple hit paradigm where oxidative stress, adipocytokines, inflammatory cytokines and an underlying condition of obesity are major players in causing NASH [4]. Though several studies have been carried out in recent years for finding plausible mechanisms for NASH causation and treatment, the role of oxidative stress, adipokine leptin and its effect on sinusoidal endothelial dysfunction and NASH progression has been unclear. NASH progression is often associated with initiation of capilarization and loss of fenestrae that may lead to ineffective sinusoidal perfusion [5]. The deficient sinusoidal drainage is strongly associated with adherence of leukocytes to sinusoidal endothelial cells and can result in expression of increased hepatic ICAM-1, VEGFR-2, Cdh5, CD34, CD31 (PECAM1), E-selectin and other molecular mediators for leukocyte extravasation and transendothelial migration [6]. Hepatic ICAM-1 and E-selectin are expressed on the endothelial cells and are included in the category of cell adhesion molecules induced by VEGF, thus helping in transendothelial migration and leukocyte infiltration [7]. The events that follow might form secondary inflammatory foci in the hepatic sinusoidal areas, thus increasing the risk of collagen deposition [8].

Leptin, an adipokine produced in the liver and the adipose tissue, is thought to contribute, in part, to NASH development in obesity through its proinflammatory actions on sinusoidal epithelial cells and Kupffer cells [9–12]. Recent lines of evidence support the role of elevated levels of leptin found in obesity in generating reactive oxygen and reactive nitrogen species and subsequent free radical formation [13]. The presence of high levels of leptin in obesity certainly makes it a prime candidate for amplifying the risk of NASH progression as both a first and second hit, which not only satisfies the two-hit hypothesis, but also is in line with the multi-hit paradigm [4]. Our own studies have demonstrated that leptin mediates the effect on NASH progression through peroxynitrite formation and Kupffer cell activation in a toxin model of NASH [14]. Leptin has been found to promote fibrosis by its effect on stellate cell proliferation [15–17]. Further leptin has been implicated in endothelial dysfunction of obesity and neovascularization in NASH [18,19]. Hepatic neovascularization and expression of vascular endothelial growth factor, a potent angiogenic factor were increased in NASH models but absent in rats that did not have leptin [18]. Endothelial dysfunction has been recently shown to be an early incidence in NASH progression [20]. Since elevated leptin has a role in endothelial dysfunction, proinflammatory and profibrotic action in mediating NASH progression, it will be important to see whether it can regulate these pathways through epigenetic modulation, especially by up regulating microRNAs.

microRNAs (miRs) are conserved, small (20–25 nucleotide) non-coding RNAs that negatively regulate expression of messenger RNAs (mRNAs) at the post-transcriptional level [21–26]. miRNAs have been found to be differentially expressed in cardiac remodeling and ischemia reperfusion injury [27]. They are also reported to be central players in anti- and profibrotic gene regulation during liver fibrosis [28]. Associations between circulating microRNAs (miR21, miR34a, miR122 and miR451) and non-alcoholic fatty liver has been documented [29]. miR21 and miR155 were found to be significantly up regulated in mice fed with a choline deficient and amino acid deficient diet (CDAA) which developed NASH and hepatocellular carcinoma [30]. miR21 has been found to target grainyhead-like 3 (Grhl3), causing its repression and this can leading to dephosphorylation of endothelial nitric oxide synthase (NOS3), a crucial mediator of endothelial function [31,32]. Based on the above literature reports, we hypothesized that adipokine leptin mediates endothelial dysfunction; inflammation and fibrosis through upregulation of miR21 and repression of target Grhl3. To study the mechanisms underlying the leptin-miR21 axis we used toxin-induced experimental NASH models (Bromodichloromethane and Carbon tetrachloride) which included oxidative stress as a second hit in an underlying condition of obesity and insulin resistance. The results showed that leptin and leptin signaling through its receptor up regulates miR21 in NASH livers. The upregulation of miR21 strongly correlated to depletion of Grhl3, decrease in NOS3 phosphorylation and increase in the protein levels of sinusoidal endothelial dysfunction markers, while leptin knockout, leptin receptor knockout or miR21 knockout mice did not show any of the described effects. We also validated our results in an accepted model of steatohepatitis and fibrosis Methionine-Choline deficient (MCD) diet that does not have an underlying condition of obesity.

## Materials and Methods

### Mouse Model

Pathogen-free, adult male mice with a C57BL/6J background (Jackson Laboratories, Bar Harbor, Maine) were used as toxin-induced models of NASH. The animals were fed with a high-fat diet (60% kcal) from 6 weeks to 16 weeks to develop diet induced obesity. All experiments were conducted at the completion of 16 weeks. Mice that contained the deleted ob/ob gene (B6.V-Lep^ob^/J) (Jackson Laboratories) (Lep KO) and another group of leptin knockout mice treated with leptin (leptin supplemented group, Lep KO+ Leptin), and the mice that contained the deleted db/db gene (B6.BKS(D)-Lepr^db^/J) (Leptin receptor knockout, Lepr KO) and mice that contained the disrupted microRNA21 gene (B6;129S6-Mir21a^tm1Yoli^/J) (miR21 KO) were fed with a high-fat diet and treated identically to DIO mice. All transgenic mice and the spontaneous knockout for leptin mice were from a C57BL6/J background. The mice were housed one in each cage in a temperature-controlled room at 23–24°C with a 12h light/dark cycle and they had ad libitum access to food and water. All animals were treated in strict accordance with the NIH Guide for the Humane Care and Use of Laboratory Animals and local IACUC standards. The experiments were approved by the institutional review board at NIEHS, Duke University and the University of South Carolina.

### Diet-induced NASH mouse model (Dietary Model)

Pathogen-free, adult male with a C57BL/6J background (wild type) fed with methionine and choline deficient (MCD) diet and were used as models for diet-induced NASH. The other set of wild type of mice were fed with methionine and choline sufficient (MCS) diet and used as a control for MCD diet-fed mice. The mice were fed with MCS or MCD diet from 8 weeks to 16 weeks and livers were collected at 1 week (MCS (1w)) or (MCD (1w)), 4 weeks (MCS (4w)) or (MCD (4w)) and 8 weeks (MCS (8w)) or (MCD (8w)) for later experiments.

### Induction of Liver Injury in Obese Mice (Toxin Model)

DIO mice at 16 weeks were administered Bromodichloromethane (BDCM) (2.0 mmoles/kg, diluted in corn oil) through the intraperitoneal route and liver tissue were collected at 24 hour post exposure (DIO+BDCM (24h)) and 48 hour post exposure (DIO+BDCM (48h)). The other group of DIO mice was exposed to BDCM as 1.0 mmole/kg, diluted in corn oil, two doses per week for one week (DIO+BDCM (1w)) and for four weeks post beginning of the toxin administration (DIO+BDCM (4w)). The other set of DIO mice at 16 weeks were administered carbon tetrachloride (CCl_4_) (60 mg/kg, diluted in corn oil) through the intraperitoneal route, two doses per week and liver tissue was collected at 1 week (DIO+CCl_4_ (1w)). High-fat diet-fed gene specific knockout (Lep KO, Lepr KO and miR21 KO) mice at 16 weeks were administered BDCM (1.0 mmole/kg, diluted in corn oil) through the intraperitoneal route. However, DIO mice treated with corn oil (diluent of BDCM) were used as control. After completion of the treatment, mice of all study groups were sacrificed for liver tissue, blood and serum for the further experiments.

The MCD model is an eight week study and shows slow chronic progression of NASH amidst fibrosis without the underlying condition of obesity and high peripheral insulin resistance. Since the whole objective of the study was to exhibit the mechanism of sinusoidal injury, it was justifiable to use different models of NASH where mir21 expression would be correlated to sinusoidal injury. The mechanisms have been worked in the toxin “two hit model of NASH” which has an underlying condition of obesity and insulin resistance. This augurs well experimentally since the induction of NASH by a second hit toxin can be controlled externally.

### Histopathology

Formalin-fixed, paraffin embedded liver tissue form study groups were cut in 5μm thick sections. Sections were deparaffinized using standard protocol and stained with picro-sirius red. Picro-sirius red staining of liver sections was carried out by using Nova ultra sirius red stain kit following manufacturer’s protocol (IHC world, Woodstock, MD) and observed under the light microscope using 20× objectives. Stained liver sections were examined for stages of fibrosis using the criteria of the NIH Non Alcoholic Steatohepatitis Clinical Research Network (NIH NASH CRN) scoring system. Stages of fibrosis were determined as 1A: mild, 1C: Portal, 2: Periportal fibrosis and 3: Bridging fibrosis.

### Western Blotting

30 mg of tissue from each liver sample was homogenized in 500 μl of RIPA buffer (Sigma Aldrich) in the presence of phosphatase and protease inhibitor (Pierce, Rockford, IL) using dounce homogenizer. Homogenate was centrifuged; the supernatant was collected for further experiments. 40 μg of protein from each sample was loaded on 4–12% bis-tris gradient gel (Invitrogen, California, USA) and subjected for SDS PAGE. Proteins were transferred to nitrocellulose membrane using precut nitrocellulose/filter paper sandwiches (Bio-Rad Laboratories Inc., California, USA) and Trans—Blot Turbo transfer system (Bio-Rad). Blots were blocked with 5% non-fat milk solution or 3% BSA (for phosphorylated proteins). Rabbit anti-mouse primary antibodies against Grhl3 (1:300), NOS3 (1:750), NOS3-p (1:750) (Santa Cruz biotechnology, Inc. Santa Cruz, CA) and β-actin (1:3000) as a reference control, were incubated overnight at 4°C. Goat anti-rabbit HRP-conjugated secondary antibody (1:6000), obtained from Abcam Inc. (Cambridge, MA) were used. Pierce ECL Western Blotting substrate (Thermo Fisher Scientific Inc., Rockford, IL) was used. The blot was developed using BioMax MS Films and cassettes (with intensifying screen, Kodak). The images were subjected to densitometry analysis using LabImage 2006 Professional 1D gel analysis software.

### Primary Liver cell culture and treatments

Primary mouse liver sinusoidal endothelial cells (LSECs) were isolated using collagenase perfusion, iodixanol density gradient centrifugation and centrifugal elutriation as previously described [33]. LSECs pooled from 12 mice were cultured on collagen coated plates in DMEM + 10% FBS. After overnight culture, medium was removed and LSECs were treated with either control, murine recombinant Leptin (20 ng/ml), LPS (1 μg/ml) or Leptin + LPS for 24 hours in DMEM. Cell lysates were collected and analyzed for miRNA expression. Primary rat liver sstellate cells and Kupffer cells were obtained from ScienceCell Research Labs, Carlsbad, CA and rat primary liver endothelial cells were obtained from Cell Biologics, Chicago, IL. Frozen cells were thawed and plated on a 6 well plate for 48 h. Following cell attachment and acclimatization, the cultures were incubated with rat recombinant leptin (100 ng/ml) and LPS (1 mg/ml) for 24h. The cells were harvested and lyzed for further processing.[33]

### Quantitative Real-Time Polymerase Chain Reaction Analysis (qRTPCR)

Gene expression (mRNA) levels in liver tissue samples were measured by two step real-time reverse transcription-polymerase chain reaction analysis. Total RNA was isolated from each 10 mg liver tissue by homogenization in 500 μl TRIzol reagent (Life Technolgies, Carlsbad, CA) according to the manufacturer’s instructions and purified with the use of RNeasy mini kit columns (Qiagen, Valencia, CA). Purified RNA (1μg) was converted to cDNA using iScript cDNA synthesis kit (Bio-rad, Hercules, CA) following the manufacturer’s standard protocol. Quantitative real-time PCR was performed with the gene specific primers using SsoAdvanced universal SYBR Green supermix (Bio-rad, Hercules, CA) and CFX96 thermal cycler (Bio-rad, Hercules, CA). Threshold Cycle (Ct) values for the selected genes were normalized against 18S (internal expression control) values in the same sample. Each reaction was carried out in triplicates for each gene and for each tissue sample. DIO mouse liver sample was used as the control for comparison with all other liver samples in the toxin model of NASH and MCS-diet-fed mouse liver sample was used as control for comparison with all other liver samples of the Dietary model of NASH. The relative fold change was calculated by the 2^−ΔΔCt^ method. The sequences for the primers used for Real time PCR are provided in given in Table 1 (mouse primers) and Table 2 (Rat primers).

**Table 1:** List of detailed primer sequences of genes used for quantitative real time PCR.

**Gene**	**Primer sequence (5’-3’)**
Leptin	Sense: GAGACCCCTGTGTCGGTTC
	Antisense: CTGCGTGTGTGAAATGTCATTG
VEGFR-2	Sense: TCTGGACTCTCCCTGCCTAC
	Antisense: TGATGCAAGGACCATCCCAC
ICAM-1	Sense: CTCAGCACTAGCACTTTGCCC
	Antisense: AACAGTTCACCTGCACGGAC
E-selectin	Sense: GTCAGCGGGACTACACACAT
	Antisense: TCTCGTCATTCCACATGCCC
VCAM-1	Sense: CGCTCAAATCGGTGACTCCA
	Antisense: TCACCTTCGCGTTTAGTGGG
Cdh5	Sense: AGGCTAGACCGGGAGAAAGT
	Antisense: CACAGTGGGGTCATCTGCAT
VEGF-α	Sense: AGGCAGACTATTCAGCGGAC
	Antisense: CCAACCTCCTCAAACCGTTG
CD34	Sense: TGGGTAGCTCTCTGCCTGAT
	Antisense: GCTGGTGTGGTCTTACTGCT

**Table 2:** List of detailed primer sequence for Rat genes.

**Gene**	**Primer sequence (5’-3’)**
RN-GRHL3	Sense: CCCCAGGTCCAAGTAAGCTG
	Antisense: CAAAGTCGTGTGTGGGTGGA
RN-VEGFR-2	Sense: AAAGAGAGGGACTTTGGCCG
	Antisense: GTCGCCACTTGACAAAACCC
RN-ICAM-1	Sense: GCCTGGGGTTGGAGACTAAC
	Antisense: CTGTCTTCCCCAATGTCGCT
RN-E-selectin	Sense: CAGCGAGGCCACATGAAATG
	Antisense: GAACACTGTACCCCTGCACA
RN-VCAM-1	Sense: TGGGGATTCCGTTGTTCTGAC
	Antisense: AGTGTGGATGTAGCCCCTTC
RN-Cdh5	Sense: TACACACAGGTGCAGAAGCC
	Antisense: GTGCAGTGTATCGTAGGGGG
RN-VEGF-α	Sense: ACTCATCAGCCAGGGAGTCT
	Antisense: GGGAGTGAAGGAGCAACCTC
RN-CD34	Sense: GAGACTCAGGGAAAGGCCAAT
	Antisense: GTTCTGTGTCAGCCACCACAT

### miR21 expression levels in liver tissues

Total miRNA was isolated from 30 mg liver tissue by homogenization in 700 μl Qiazol reagent (Qiagen, Valencia, CA) according to the manufacturer’s instructions and purified with the use of miRNeasy mini kit columns (Qiagen, Valencia, CA). Purified miRNA (1μg) was converted to cDNA using miScript cDNA synthesis kit (Qiagen, Valencia, CA) following the manufacturer’s standard protocol. Quantitative real-time PCR was performed with the gene specific primers using miScript SYBR Green PCR master mix (Qiagen, Valencia, CA) and CFX96 thermal cycler (Bio-rad, Hercules, CA). Threshold Cycle (Ct) values for the selected genes were normalized against RNU6-2 (internal expression control) values in the same sample.

### Immunofluorescence microscopy

Paraffin-embedded liver tissue from DIO, DIO+BDCM (1w), Lep KO (1w), Lepr KO (1w), MCS (4w), MCD (4w), miR21 KO+BDCM (1w) and miR21 KO+MCD (4w) groups was cut into 5 μm thick sections. Each section was deparaffinized using standard protocol. Briefly, sections were incubated with xylene twice for 3 min, washed with xylene:ethanol (1:1) for 3 min and rehydrated through a series of ethanol (twice with 100%, 95%, 70%, 50%), twice with distilled water and finally rinsed twice with phosphate buffered saline (PBS). Epitope retrieval of deparaffinized sections was carried out using epitope retrieval solution and steamer (IHC-world, Woodstock, MD) following manufacturer’s protocol. The anti-mouse primary antibodies (i) anti-VEGFR-2 was purchased from AbCam Inc. (Cambridge, MA), (ii) anti-ICAM-1, and (iii) anti-E-selectin were purchased from Santa Cruz biotechnology, Inc. (Santa Cruz, CA), and used in 1:150 dilutions. Species-specific anti-IgG secondary antibody conjugated with Alexa Fluor 633 (Invitrogen, California, USA) was used to localize the sinusoidal endothelial dysfunction biomarker proteins. Sections were mounted in ProLong gold antifade reagent with DAPI. Images were taken under 20× objectives using Olympus BX51 microscope.

### Statistical Analyses

All experiments were repeated three times with 3 mice per group (N = 3; data from each group of mice was pooled). The statistical analysis was carried out by analysis of variance (ANOVA) followed by the Bonferroni posthoc correction for intergroup comparisons. Quantitative data from Western blots as depicted by the relative intensity of the bands were analyzed by performing a student’s t test. P<0.05 was considered statistically significant.

## Results

### NASH progression is associated with increased expression of hepatic leptin

**Figure 1:**
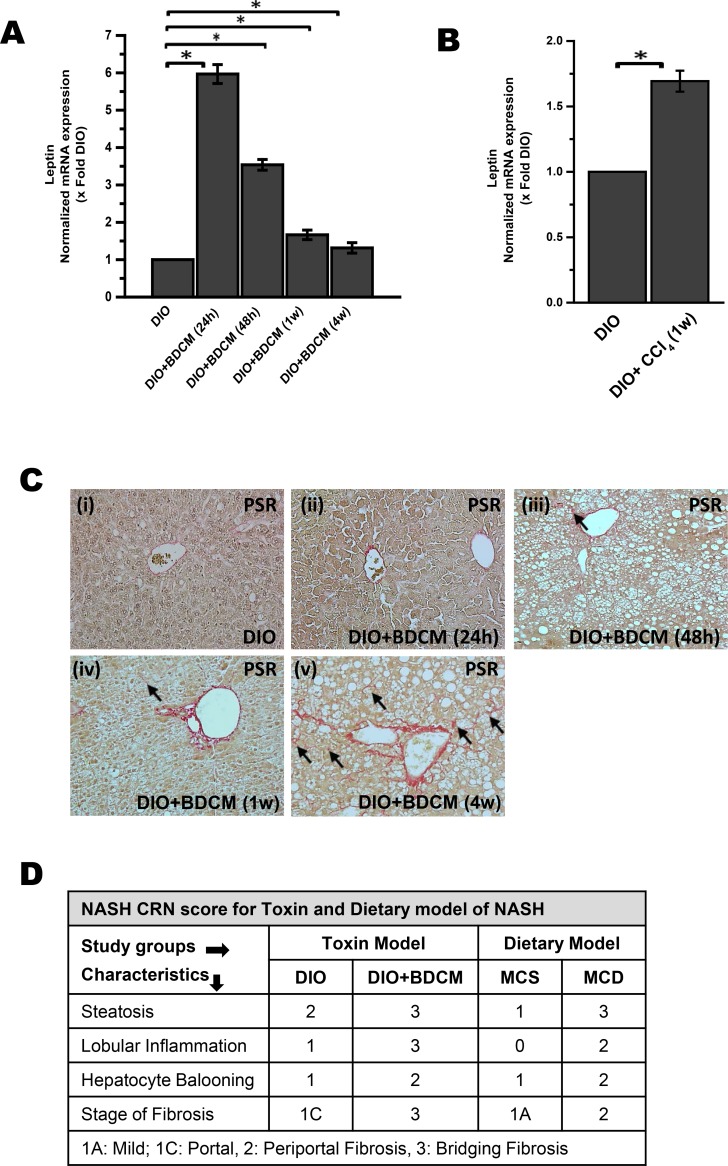
Increased hepatic leptin is associated with NASH progression in obesity. qRTPCR analysis of hepatic leptin mRNA expression in two toxin model of NASH. *A*. Bromodichloromethane (BDCM) model: Y-axis represents fold of leptin mRNA expression in DIO, DIO mice exposed with BDCM for 24h, for 48h, for 1week and for 4 weeks post BDCM exposure. *B*. Carbon tetrachloride (CCl_4_) model: Y-axis represents fold of leptin mRNA expression in DIO and DIO mice exposed with CCl_4_ for 1w. n = 3, P<0.05 is considered statistically significant (*). *C*. Picro-sirius red (PSR) staining of liver sections of DIO, DIO+BDCM at 24h, DIO+BDCM at 48h, DIO+BDCM at 1w and DIO+BDCM at 4w post BDCM exposure; 20× images (n = 3). Black arrowhead depicts macro and micro vesicular fibrosis. *D*. Stages of fibrosis of stained liver sections from two different model of NASH (toxin and dietary model) were reviewed using the criteria of the NIH Non Alcoholic Steatohepatitis Clinical Research Network (NIH NASH CRN). Table depicts the NASH CRN scores for DIO, DIO+BDCM (toxin model) and MCS, MCD (Dietary model).

Association of leptin with endothelial dysfunction in obesity is known [19]. It has been shown that leptin increased NO production concomitant to cytotoxic peroxynitrite production [19]. This led to a decrease in L-arginine concentration and caused uncoupling of NOS3 [19]. Though there are studies which show the mechanism of leptin based on NOS3 uncoupling, we hypothesized that the proinflammatory role of hepatic leptin might be crucial for endothelial dysfunction by up regulating miR21, a microRNA that has a pronounced role in inflammation. Leptin promotes hepatic fibrogenesis through upregulation of Transforming growth factor beta in Kupffer cells and sinusoidal endothelial cells [34,35]. Further, leptin facilitates proliferation and prevents apoptosis of hepatic stellate cells [9]. Obesity-induced leptin plays a crucial role in NASH progression via enhanced response to endotoxin [36]. Our own laboratory investigations have shown that in livers of experimental models of NASH, higher levels of oxidative stress-induced leptin caused inflammation, Kupffer cell activation and CD8+CD57+T cells proliferation and played an important role in the development and progression of NASH [14,37,38]. In the present study we show that leptin mRNA expression had a time-dependent increase post toxin exposure in steatotic liver. Leptin mRNA expression increased 6 fold following BDCM exposure at 24h and stayed at higher levels as compared to DIO group (Fig. 1A) (P<0.05). Leptin mRNA expression was 1.6 fold higher in the DIO+CCl_4_ group at 1 week (1w) post exposure (Fig. 1B) (P<0.05).The increased levels of leptin correlated well with progression of steatohepatitis as shown by picro-sirius red staining and NASH CRN scores at 4w post “second hit” BDCM exposure. Staining for fibrosis as shown by picro-sirius red staining was higher in DIO+BDCM group at 4w as compared with DIO (Fig. 1C (i) and (v)). Fibrosis was not comparably different at 24h, 48h or at 1w post BDCM exposure (Fig. 1C (ii, iii and iv)). NASH CRN scores showed increased hepatocyte necrosis, ballooning and bridging fibrosis in DIO+BDCM group as compared to DIO group only (Fig. 1D). Previously, we reported that leptin mRNA levels were significantly higher in MCD diet-fed mice [14]. The results showed that apart from the high circulatory leptin that is present in an underlying condition of obesity, a progressive phase of NASH is accompanied by an even higher leptin in the liver.

### NASH progression results in time-dependent increase in miR21 and concomitant repression of its target protein Grhl3

**Figure 2:**
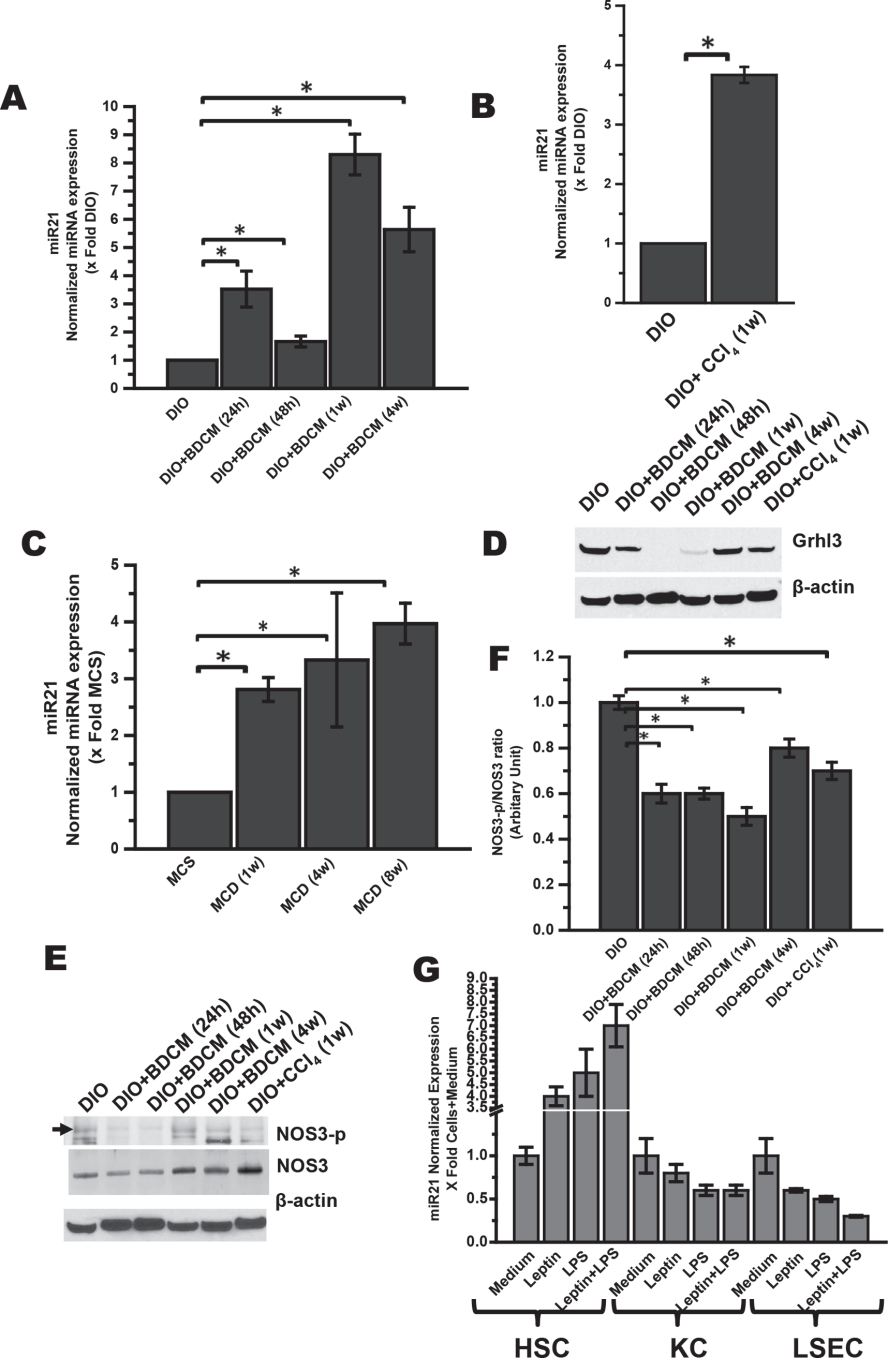
NASH progression mediated by miR21 expression and concomitant repression of Grhl3 protein. A qRTPCR analysis of hepatic miRNA21 expression in toxin and diet model of NASH *A*. BDCM model: Y-axis represents fold of miR21 expression in DIO, DIO mice exposed with BDCM for 24h (DIO+BDCM (24h)), for 48h (DIO+BDCM (48h)), for 1week (DIO+BDCM (1w)) and for 4 weeks (DIO+BDCM (4w)). *B*. CCl_4_ model: Y-axis represents fold of miR21 expression in DIO and DIO mice exposed with CCl_4_ for 1w. *C*. MCD model: Y-axis represents fold of miR21 expression in MCS (control for MCD diet), mice fed with MCD diet for 1 week (MCD (1w)), for 4 weeks (MCD (4w)) and for 8 weeks (MCD (8w)). n = 3, P<0.05 is considered statistically significant (*). Results showed a time-dependent increase in miR21 levels that correlates with NASH histopathology. *D*. Western blot analysis of Grhl3 (target of miR21) protein levels in liver homogenate from DIO, DIO+BDCM (24h), DIO+BDCM (48h), DIO+BDCM (1w), DIO+BDCM (4w) and DIO+CCl_4_ (1w) mice groups. Corresponding β-actin levels are shown in the lower panel. *E*. Western blot analysis of endothelial nitric oxide synthase (NOS3) and phosphorylated NOS3 (NOS3-p) in liver homogenate from DIO, DIO+BDCM (24h), DIO+BDCM (48h), DIO+BDCM (1w), DIO+BDCM (4w) and DIO+CCl_4_ (1w) mice groups. Corresponding β-actin levels are shown in the lower panel. *F*. Levels of phosphorylated NOS3 (NOS3-p) protein normalized against respective NOS3 levels and β-actin levels (NOS3-p/NOS3 ratio) were plotted. Y-axis represent arbitrary unit of NOS3-p/NOS3 ratio of mice groups from both BDCM and CCl_4_ model. P<0.05 is considered statistically significant (*). G. mir21 expression in liver primary cells incubated with LPS and leptin. Isolated cells were incubated for 24h and cell lysates were analysed for mir21 expression using Qrtpcr. P<0.05 is considered statistically significant.

The relatively recent discovery of microRNAs (miRNAs) has exposed an extra layer of gene expression regulation that affects many physiological and pathological processes of diseases [39]. Alisi et al, showed in a miRNome analysis the potential involvement of novel determinants (miRNAs and proteins) in the molecular pathogenesis of diet-induced NAFLD [40]. miR21 has been shown to be up regulated in a very few studies in NASH [30]. However, the upstream modulators of miR21 regulation and its biological effect on specific etiologies in NASH have not been explored to its potential. A detailed miRNA array analysis done by a commercial vendor identified several miRNAs including miR21 to be up regulated in our models of NASH. A detailed analysis of target proteins by available literature identified among many other proteins Grhl3 to be a probable target of miR21. To show the role of NASH progression in inducing miR21 and its principle target protein Grhl3, qRTPCR analysis was carried out at different time points and in three experimental models of NASH, namely, the two toxin models of NASH in a background of obesity and insulin resistance (DIO+BDCM and DIO+CCl_4_), and the dietary model of NASH (MCD diet). Results showed that there was a time-dependent increase in miR21 levels (Fig. 2A, 2B and 2C) that correlated well with NASH histopathology and picro-sirius red staining (Fig. 1C and 1D). Results also showed that miR21 levels increased significantly at 24h post BDCM exposure followed by a slight decrease at 48h (Fig. 2A). Levels of this particular miRNA increased significantly (8 fold) at 1w post BDCM exposure and stayed at a higher level at 4w, as compared to DIO only group (Fig. 2A) (P<0.05). There was a 3.8 fold increase in the miR21 levels in CCl_4_-induced steatohepatitis in obese mice as compared to DIO only group (Fig. 2B) (P<0.05). MCD diet-fed mice had a significant increase of miR21 at 1w post diet exposure as compared to MCS control diet-fed mice (Fig. 2C) (P<0.05). Interestingly MCD group at 4w showed a 3 fold increase of miR21 as compared to the control MCS group and stayed at a higher level throughout the duration of the study (8w) (Fig. 2C) (P<0.05). Western blot analysis of miR21 target protein Grhl3 showed a sharp decrease in both DIO+BDCM group and DIO+CCl_4_ group as compared to DIO only group (Fig. 2D). Western blot band analysis was not performed in this figure because of the higher magnitude of repression of the protein at 1w. miR21 upregulation and its corresponding targeting of Grhl3 is known. Grhl3 regulates the PI3AKT-NOS3 (endothelial nitric oxide synthase) pathway [31]. It is of paramount importance that miR21 upregulation in NASH would have a significant impact on the target proteins, that would in turn affect positively the NASH progression. Results showed that Grhl3 was decreased during the entire study period except at the termination stage of the toxin group model of NASH. The results assume significance since Grhl3 has been shown to phosphorylate NOS3 [31,41]. NOS3 activation is significant for increased nitric oxide bioavailability, crucial for endothelial function [20,42]. NASH is associated with sinusoidal endothelial dysfunction and has been shown to be an early event in pathogenesis [20]. Results also showed that, there was a decrease in NOS3 phosphorylation as shown by decreased NOS3-p/NOS3 (eNOS-p/eNOS) ratio in DIO+BDCM group at 24h, 48h, and 1w time points as compared to DIO only group as shown by western blot analysis and corresponding ratio of phosphorylation based on the differential immunoreactivity against the phosphorylated NOS3 antigen present in the liver tissue homogenates (Fig. 2E and 2F) (P<0.05). Interestingly, NOS3-p/NOS3 ratio in DIO+BDCM group was comparable to DIO group at 4w (Fig. 2F). The ratio was calculated based on the anticipation that it might be correlated well with NASH developmental stages where sinusoidal endothelial dysfunction might be detected (1w post BDCM exposure initiation).

### miR21 expression and its target Grhl3 in NASH progression are dependent on the presence of leptin

**Figure 3:**
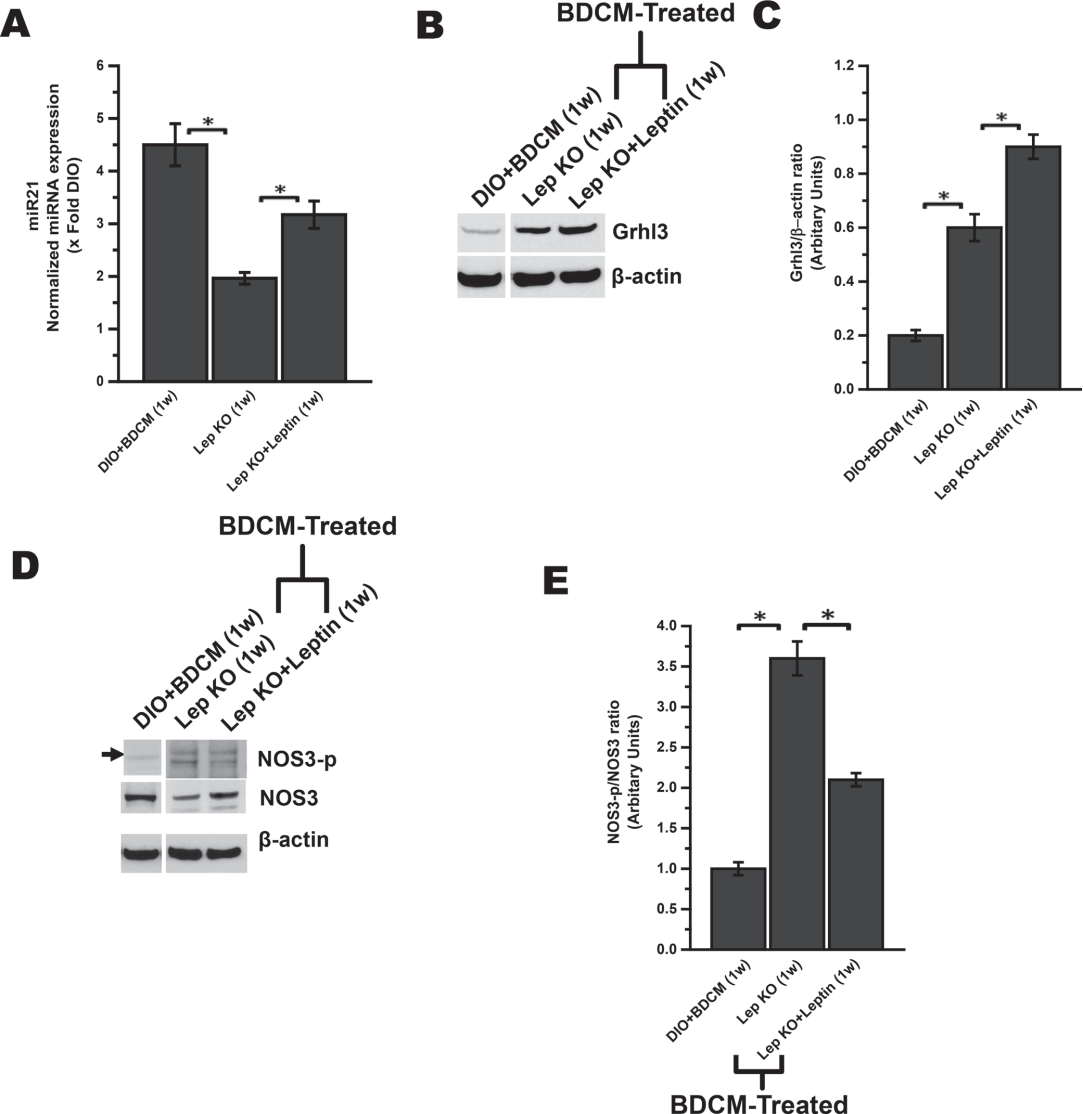
Increased miR21 expression, subsequent decreased expression of Grhl3 protein and NOS3 phosphorylation is mediated by leptin. *A*. miR21 expression as measured by quantitative real-time PCR in DIO mice exposed with BDCM (DIO+BDCM (1w)), ob/ob gene deficient mice exposed with BDCM (Lep KO (1w)) and ob/ob gene deficient mice supplemented with leptin exposed with BDCM (Lep KO+Leptin (1w)). P<0.05 is considered statistically significant (*) *B*. Western blot analysis of Grhl3 in liver homogenates of BDCM exposed DIO mice (DIO+BDCM (1w)), mice that lacked the ob/ob gene and exposed with BDCM (Lep KO (1w)) and Lep KO supplemented with leptin and exposed with BDCM (Lep KO+Leptin (1w)). *C*. Column graph depict the band quantification analysis of Grhl3 protein with corresponding β-actin as shown in lower levels of fig B. *D*. Western blot analysis of phosphorylated NOS3 (NOS3-p) and NOS3 in DIO+BDCM (1w), Lep KO (1w) and Lep KO+Leptin (1w) mice group. *E*. NOS3-p/NOS3 ratio (indication of vascular endothelium function) was plotted after band quantification and normalization against respective β-actin. Y-axis represent arbitrary unit of NOS3-p/NOS3 ratio of DIO+BDCM (1w), Lep KO (1w) and Lep KO+Leptin (1w) mice groups. P<0.05 is considered statistically significant (*). #Band image of DIO+BDCM (1w) has been cropped from the same immunoblot image and placed separately in both fig B and D due to presence of other mouse group in between the DIO+BDCM (1w) and Lep KO (1w) lane which is not explained in this manuscript. The cropped images of the blot are separated by a distinct blank space to show the non-continuity of the image.

miR21 induction has been reported in inflammatory diseases [43]. miR21 induction by NF-κB binding to its promoter has been shown in in vitro cell culture conditions [44]. Previous studies from our laboratory has shown that leptin contributes significantly in upregulation of oxidative stress, inflammation and Kupffer cell activation [14]. Leptin’s role as a proinflammatory adipokine has been well established [45,46]. To prove the role of leptin in inducing miR21, we used two different mouse models. ob/ob mice (Lep KO), a spontaneous knockout of leptin, and recombinant leptin supplementation in ob/ob mice (Lep KO +Leptin) were used to find the role of leptin in inducing miR21. Results showed that miR21 expression was significantly decreased in ob/ob mice (Lep KO) exposed to the toxins for induction of NASH as compared to wild type DIO mice (Fig. 3A) (P<0.05). Leptin supplementation to ob/ob mice (Lep KO+ Leptin) significantly increased the miR21 levels as compared to Lep KO group (Fig. 3A) (P<0.05). In parallel, leptin administration in ob/ob mice restored the miR21 levels found in DIO mice that had NASH, suggesting the requirement of leptin in induction of miR21. miR21 has been shown to target Grhl3 in this study and many other proteins in inflammatory conditions to cause their repression as a post-transcriptional regulatory mechanism [43]. Since miR21 targeted Grhl3 in our studies, we studied the levels of Grhl3 in ob/ob, and leptin supplemented group to establish the direct link between leptin and miR21 linked post-transcriptional modifications to its targets. Results showed that ob/ob (Lep KO) mice that were injected with the toxins for induction of NASH had significantly elevated Grhl3 levels as compared to DIO+BDCM mice that had NASH symptoms at 1w post toxin exposure (Fig. 3B) as shown by western blot analysis and band quantification (3 fold increase) (Fig. 3C) (P<0.05). Surprisingly, leptin supplementation as shown by Lep KO+Leptin group however had a marginal increase in the levels of Grhl3 as compared to ob/ob mice (Lep KO group) (Fig. 3C) (P<0.05). These results suggested that leptin was critical for repression of this protein and a miR21 dependent mechanism might be responsible for a post-transcriptional regulation of Grhl3. To study the effect of Grhl3 repression and its modulation by adipokine leptin, NOS3 phosphorylation was estimated. Using immunoblot assay and subsequent analysis of the immunoreactive bands, it was shown that Lep KO group had significantly less NOS3 phosphorylation (Fig. 3D). Analysis of the immunoreactive bands of NOS3 phosphorylation and the ratio of NOS3-p/NOS3 showed a 3.6 fold increase in the absence of leptin (Fig. 3E) (P<0.05). Supplementation of recombinant leptin to leptin KO mice significantly decreased NOS3 phosphorylation as shown by western blot analysis (Fig. 3D) and band quantification (Fig. 3E). A 1.5 fold decrease in NOS3-p/NOS3 ratio was observed in the leptin supplemented group as compared to Lep KO group (Fig. 3E) (P<0.05). To elucidate the cellular basis of the leptin-mediated mir21 increase, rat primary liver sinusoidal endothelial cells, hepatic stellate cells and Kupffer cells were incubated with or without leptin for 24 h. Results showed that LSECs and Kupffer cells showed a down regulation of mir21 while hepatic stellate cells showed a six fold increase in the mir21 expression as shown in S1A Fig. GRHL3 protein levels showed a concomitant decrease in stellate cells while the protein levels were unchanged in other cell types as shown in S1A-B Fig. This might suggest an indirect role of downstream GRHL3 based signaling, albeit as a paracrine modulator in other cell types.

### Presence of leptin is required for increase in markers of sinusoidal endothelial injury

**Figure 4:**
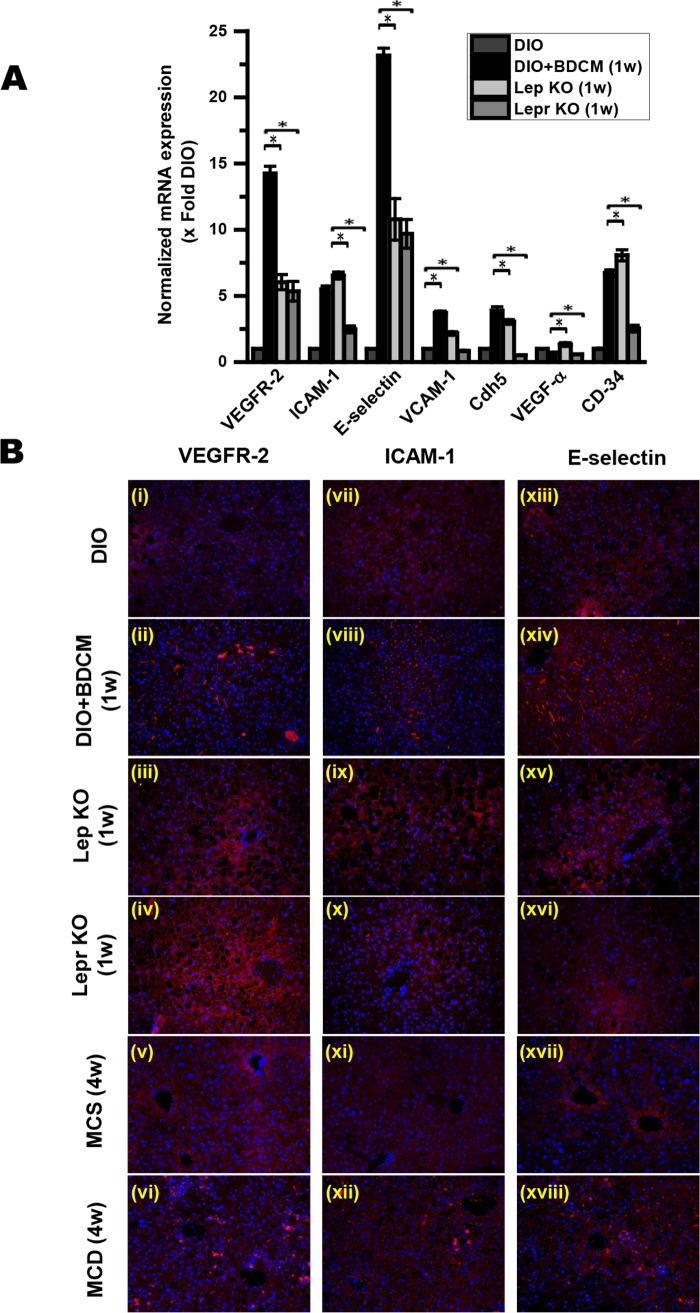
Sinusoidal endothelial dysfunction (SED) in NASH progression is mediated by leptin. *A*. mRNA expression of sinusoidal endothelial dysfunction biomarkers (VEGFR-2, ICAM-1, E-selectin, VCAM-1, Cadherin 5 (Cdh5), VEGF-α and CD34) as measured by quantitative real-time PCR in DIO+BDCM (1w), ob/ob gene deleted mice (Lep KO (1w)) and db/db gene deleted mice (Lepr KO (1w)). Y-axis shows fold of mRNA expression of SED biomarkers normalized against DIO only groups. *B*. Immunofluorescence images for localization of SED biomarkers (VEGFR-2, ICAM-1 and E-selectin) from liver sections of both toxin model (DIO, DIO+BDCM (1w), Lep KO (1w), Lepr KO (1w)) and dietary model (MCS (4w) and MCD (4w)) of NASH.

Leptin has been shown to be involved in the sinusoidal endothelial dysfunction in obesity and neovascularization in NASH [18,19]. Sinusoidal endothelial dysfunction in NASH is a crucial early event in its progression [20]. To prove the role of leptin and leptin signaling through its receptor in sinusoidal endothelial dysfunction in our model of NASH, we studied the key biomarkers of endothelial dysfunction and injury (mRNA expression of VEGFR-2, ICAM-1, E-selectin, VCAM-1, Cadherin-5 (Cdh5), VEGF-α and CD34, using ob/ob (Lep KO) and db/db (Lepr KO) mice. We included a new experimental group that contained mice which had a spontaneous mutation of the db/db gene (gene that encodes the leptin receptor). mRNA expression of VEGFR-2, ICAM-1, E-selectin, VCAM-1, and Cdh5 were significantly decreased in ob/ob (Lep KO) and db/db (Lepr KO) mice as compared to DIO +BDCM group mice that had NASH (Fig. 4A) (P<0.05). To prove the sinusoidal localization of the biomarkers (VEGFR-2, ICAM-1 and E-selectin), immunofluorescence microscopy was carried out on liver slices from DIO, DIO+BDCM, ob/ob and db/db mice. Results showed that red fluorescent staining (Alexa Fluor 633) was markedly increased in the sinusoids of DIO+BDCM livers that had NASH symptoms, while ob/ob and db/db mouse livers had significantly decreased staining in the sinusoids. In the present experiments we also studied the sinusoidal immunoreactivities of the above markers in MCD diet-induced NASH. Results showed that MCD diet-fed mice had increased VEGFR-2, ICAM-1 and E-selectin immunoreactivity as compared to the corresponding MCS diet-fed controls at 4w (Fig. 4B). The 4w time point is an early stage of NASH development in this model. Decreased immunoreactivity in Lep KO and Lepr KO mice suggested strongly that leptin is required for the upregulation of these sinusoidal endothelial dysfunction biomarkers in NASH.

### miR21 knockout mice do not show decreased Grhl3 and NOS3 phosphorylation and sinusoidal endothelial dysfunction in toxin-exposed and MCD diet-fed NASH models

**Figure 5:**
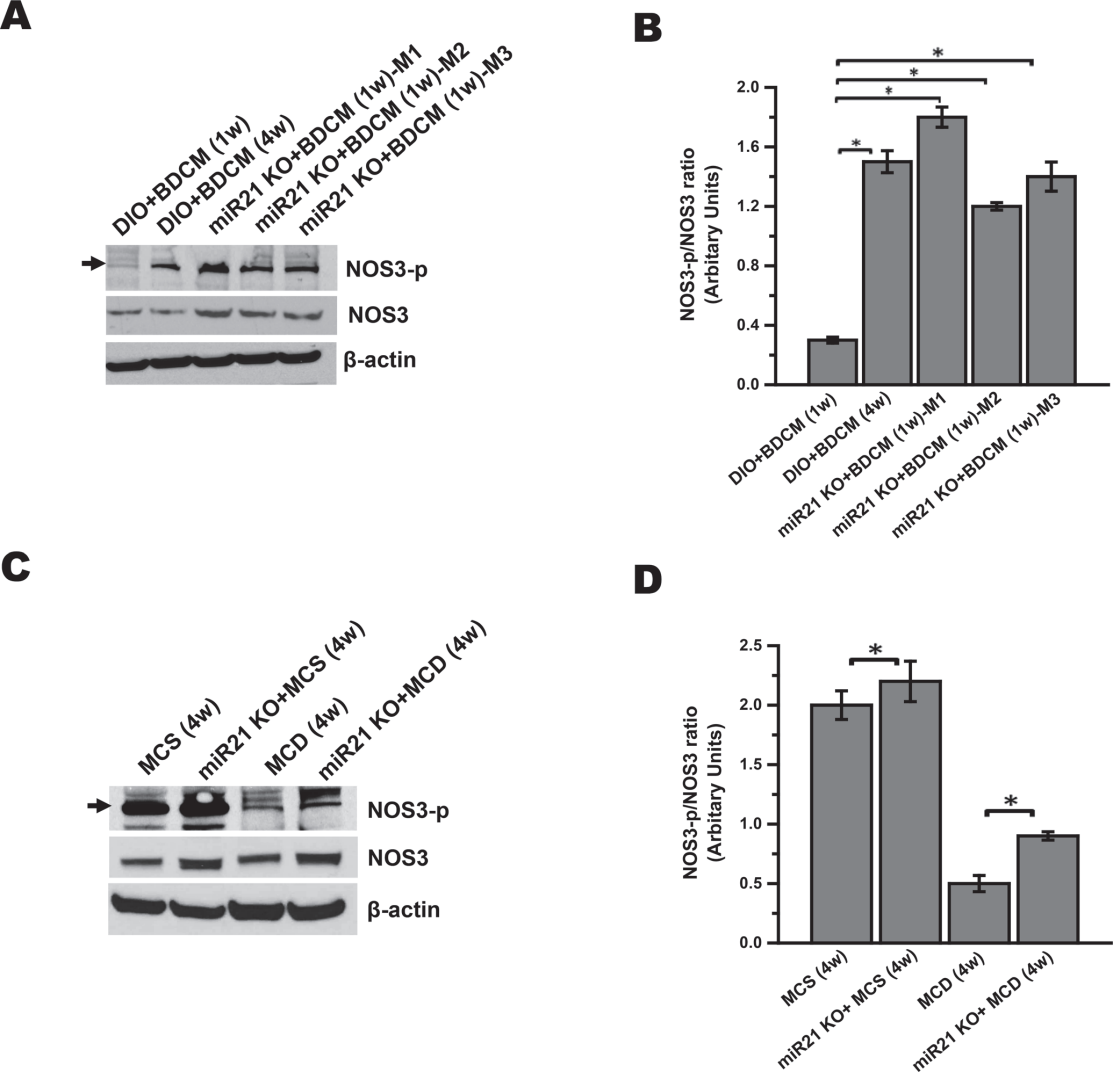
miR21 play a key role in leptin signaling of sinusoidal endothelial dysfunction. *A* and *C*. Phosphorylation of endothelial nitric oxide synthase (NOS3) is the key event in endothelial function. To access levels of NOS3 phosphorylation in liver homogenate, western blot analysis was carried out for phosphorylated NOS3 (NOS3-p) and native NOS3 protein. The mice groups for toxin model (fig. A) are DIO+BDCM (1w), DIO+BDCM (4w) and 3 individual miR21 KO mice (M1, M2 and M3) and the mice groups for dietary model (fig. C) are MCS (4w), miR21 KO fed with MCS diet (miR21 KO+MCS (4w)), MCD (4w) and miR21 KO mice fed with MCD diet (miR21 KO+MCD (4w)). The corresponding β-actin levels are shown in the lower panel. *B* and *D*. Levels of phosphorylated NOS3 (NOS3-p) protein normalized against respective NOS3 levels and β-actin levels (NOS3-p/NOS3 ratio) were plotted. Y-axis represent arbitrary unit of NOS3-p/NOS3 ratio of mice groups from both BDCM (fig. B) and diet model (fig. D) of NASH. P<0.05 is considered statistically significant (*).

**Figure 6:**
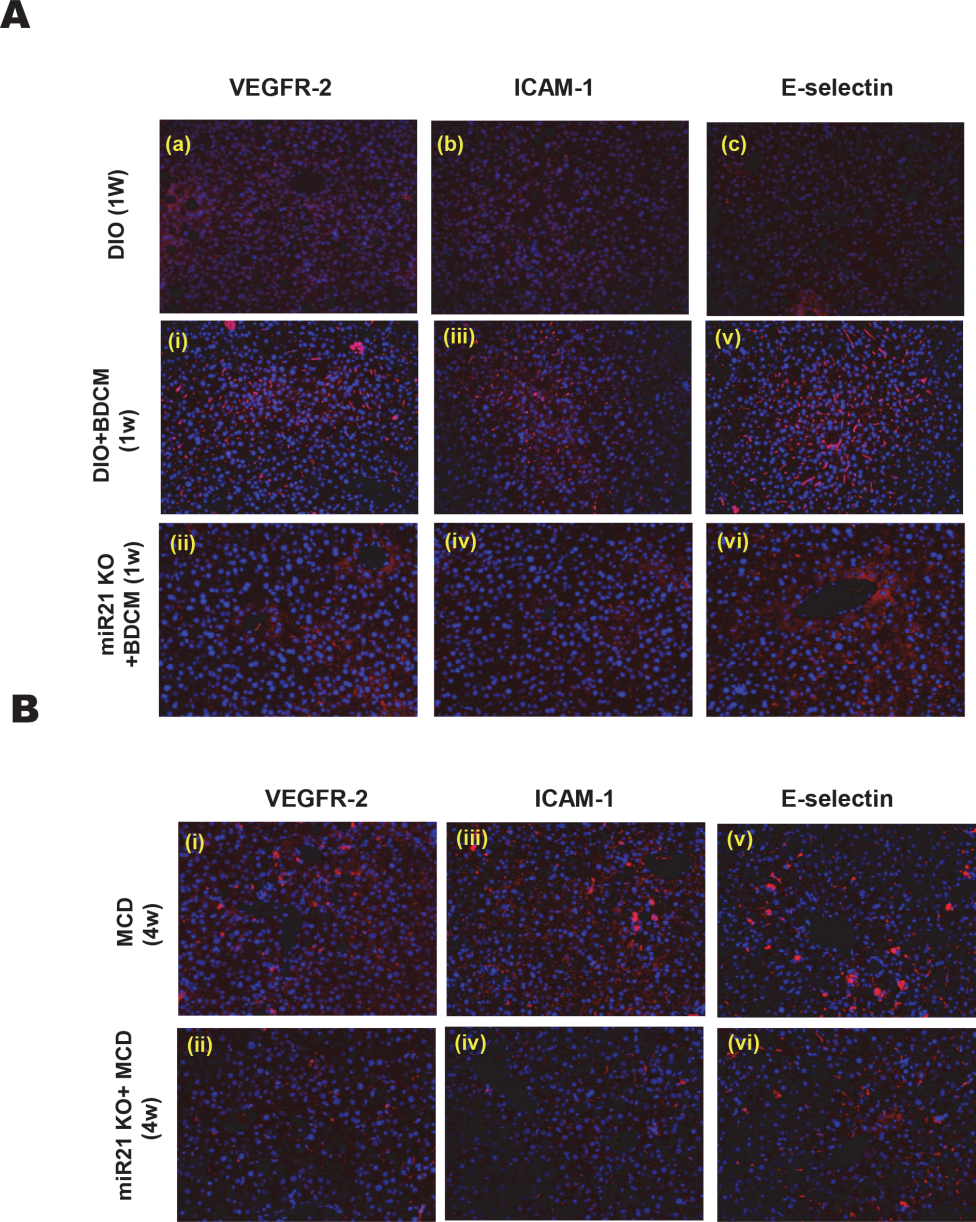
miR21 mediated sinusoidal endothelial dysfunction is the early event in NASH progression. *A* and *B*. Immunofluorescence images for localization of sinusoidal endothelial dysfunction biomarkers (VEGFR-2, ICAM-1 and E-selectin) from liver sections of toxin model groups (fig. A) DIO+ BDCM (1w) and miR21 KO+BDCM (1w) and dietary model (fig. B) MCD (4w) and miR21 KO+MCD (4w). Immunoreactivity with red dots shows the localization of SED biomarkers.

Having established the role of leptin in inducing miR21 and its concomitant repression of miR21 target proteins, it was important to study the role of leptin induced miR21 in causing sinusoidal endothelial dysfunction in our models of NASH. To prove the involvement of leptin induced miR21 in vivo, we used miR21 knockout mice that were co-exposed with a high fat diet and hepatotoxin BDCM. Similarly, mice with null mutation of miR21, fed with a MCD diet were also included. Since the establishment of miR21 as a key player in sinusoidal endothelium dysfunction was crucial for NASH progression, we chose to include the dietary model of NASH that also showed significant fibrosis without obesity. Results showed that miR21 KO mice did not have decreased GRHL3 protein as shown in S3 Fig. NOS3 phosphorylation was elevated in miR21 KO mice (individual blot data from 3 mice) as compared to DIO+BDCM group at 1w (Fig. 5A). Results also showed that miR21 knockout mice had significantly increased NOS3-p/NOS3 (eNOS-p/eNOS) ratio as compared to the DIO mice that were exposed to the toxin BDCM (DIO+BDCM) (Fig. 5B) (P<0.05). The increase observed was >4 fold in each of the mouse studied (Fig. 5B). The studies with miR21 KO mice were further extended to MCD model of NASH. Results showed that there was a 2 fold increase in NOS3-p/NOS3 ratio in miR21 KO mice as compared to wild type MCD diet-fed mice at 4w (Fig. 5C and 5D) (P<0.05). The above results suggested that leptin induced miR21 was critical for NOS3 phosphorylation that had a significant role in NOS3 bioactivity. miR21 knockout mice in both models of NASH were also studied for the expression of endothelial biomarkers VEGFR-2, ICAM-1 and E-selectin and their corresponding localization patterns by immunofluorescence microscopy. Results showed that protein expression of VEGFR-2, ICAM-1 and E-selectin, measured by the immunoreactivity of these markers in liver slices were significantly decreased in miR21 knockout mice as compared to DIO mice co-exposed to high fat and BDCM for NASH induction at 1w post initiation of toxin administration (Fig 6A). The data were also compared with DIO group as shown in panels a, b and c. miR21 KO mice in MCD model of NASH also showed a similar decrease in sinusoidal endothelial dysfunction and injury markers expression at 4w, that might be crucial for NASH progression (Fig. 6B). Further, there was an observable and marked decrease in sinusoidal staining for these biomarkers, suggesting that miR21 was at least in part involved in causing sinusoidal endothelial dysfunction in NASH.

## Discussion

Our studies for the first time showed the role of leptin-induced miR21 in significantly contributing to the early sinusoidal endothelial injury that is recently thought to contribute to the more progressive phases of NASH. Leptin-induced miR21 caused sinusoidal endothelial dysfunction primarily by repressing Grhl3, a protein that has a role in phosphorylating NOS3 and increasing NO bioavailability [31]. Further, we also show that miR21 KO mice are protected from sinusoidal endothelial dysfunction primarily by increased nitric oxide bioavailability through increased NOS3 phosphorylation. The above described results related to miR21 have relevance to the fact that sinusoidal endothelial dysfunction precedes inflammation and might play a significant role in the events of inflammation and fibrosis that follow in NASH developmental process. The fact that miR21 KO mice show decreased sinusoidal endothelial dysfunction markers can be probed further for greater understanding of inflammation and fibrosis in NASH.

Obesity is shown to have a state of leptin resistance where there is hyperleptinemia [47]. We have reported, in our earlier studies, that the “second hit” of toxins which help transformation from benign steatosis to steatohepatitis without the involvement of alcohol, caused a secondary rise in hepatic leptin at both mRNA and protein levels [14]. Our results in this study further confirmed the higher leptin mRNA levels in the NASH liver at 24h, 48h and 1w time points following “second hit” and this correlated well with higher fibrosis in experimental setting of NASH in rodents (Fig. 1). Leptin significantly contributed to peroxynitrite mediated Kupffer cell activation and inflammation in NASH [14]. Leptin also has been shown conclusively to aid in stellate cell activation and fibrosis [16,17]. We thus argued that leptin, being a proinflammatory adipokine, might play a significant role in causing sinusoidal endothelial dysfunction and disrupted microvasculature in NASH. microRNAs are believed to be central players in anti- and profibrotic gene expression in liver fibrosis [28]. Sheedy et al. described miR21 as a central player in the inflammatory response [44]. Having examined leptin’s role in Kupffer cell activation, NADPH oxidase activation and IL-1β release in experimental models of NASH, we studied the role of increased leptin in miR21 upregulation. Our results showed that hyperleptinemia was associated with significant increases in hepatic miR21 expression in an earlier time point, (one week into the study) in the toxin model of NASH and had an elevated level of the microRNA during the entire course of the study in MCD model of NASH (Fig. 2). Upregulation of miR21 has been associated with inflammation, primarily by its effect on different target proteins [43]. It is worth mentioning that miRNAs can bind to the promoter regions of specific genes that code for functionally relevant proteins involved in regulating inflammation [28]. Studies reported earlier show that PTEN, Grhl3, PPARα, cyclinD1 and SMAD7 are few of the several proteins that are targets of miR21 [28,32,43,48,49]. Grhl3 is a recently discovered protein with a role of affecting NOS3 phosphorylation [31]. NOS3 phosphorylation is an essential step for activating NOS3 activity, that is significant for nitric oxide release, and regulation of NOS3 cycle [50]. Our studies showed that Grhl3 levels were significantly repressed in experimental NASH (Fig. 2D) at earlier time points of 48h and 1w. The decreased protein levels of Grhl3 correlated well with the decreased NOS3 phosphorylation in those time points (Fig. 2E and F), signifying the importance of miR21-induced Grhl3 suppression being crucial for NO bioavailability in NASH. The results of higher hepatic leptin and its correlation with higher miR21 and repression of miR21-target protein, Grhl3, in experimental NASH led us to probe the direct role of leptin and its downstream signaling in our models of NASH.

Leptin knockout mice did not show increased miR21 levels at the same time points as mice that had NASH symptoms (DIO+BDCM group) (Fig. 3A). Leptin knockout mice were only used to simulate the absence of leptin in vivo, though such a state never exists in obesity or NASH. To overcome a condition of complete leptin absence in the leptin knockout mice, we administered recombinant leptin in these mice in an attempt to recreate a hyperleptinemic condition. Leptin supplementation into mice that did not have leptin showed a significantly increased miR21 levels, and decreased NOS3 phosphorylation (NOS3-p/NOS3 ratio of 2.1) (Fig. 3D and 3E) as compared to leptin knockout mice, thus firmly establishing that leptin induced miR21-mediated NOS3 phosphorylation, at least in part, plays a role in the sinusoidal endothelial dysfunction that might result from decreased NO bioavailability due to decreased NOS3 phosphorylation. Interestingly leptin supplementation did not significantly alter Grhl3 protein levels as compared to leptin KO mice (Fig. 3B), though there was a significant decrease in NOS3 phosphorylation (Fig. 3D). This might be due to an existence of a yet to be discovered Grhl3 protein mediated downstream signaling cascade which might also be regulated by miR21.

To elucidate the cellular basis of endothelial injury in the liver we conducted in vitro cell-based experiments with isolated rat primary liver sinusoidal endothelial cells (LSECs), hepatic stellate cells and Kupffer cells. Based on their co-existence and cross talk in the sinusoids, we chose to investigate all three cell types. Our results of stellate cells being the primary cell type with leptin-induced mir21 upregulation and its paracrine regulation of sinusoidal injury in Kupffer cells and LSECs further strengthen the importance of the cell type in modulating NASH pathophysiology as shown in S1 and S2 Figs. Future studies regarding sinusoidal injury and its regulation by the larger epigenome in the liver can be targeted in the stellate cells.

Our results with leptin KO mice and mice that did not have the leptin receptor showed significant decrease in both mRNA and protein levels of VEGFR-2, ICAM-1 and E-selectin as compared to DIO+BDCM group (Fig. 4). Endothelial dysfunction in liver sinusoidal endothelial cells (LSECs) reduces vasodilatory functions by nitric oxide, and augments vasoconstriction. This contributes to an increased intrahepatic vascular resistance and develops portal hypertension [51,52]. In vivo hemodynamic studies evaluating the sinusoidal perfusion and examination of liver microvasculature remain the mainstays of investigating liver sinusoidal endothelial dysfunction [20]. However, we chose to analyze the molecular markers in the liver sinusoidal endothelial cells that have been shown by numerous studies to be directly related to endothelial dysfunction and inflammation which follows soon after [53–55]. Thus a decreased mRNA and protein levels of VEGFR-2, ICAM-1 and E-selectin in the sinusoids of Leptin KO mice and mice that have a defective leptin downstream signaling, as shown by immunofluorescence microscopy reflect in part the role of hepatic leptin and probably miR21 in sinusoidal inflammation and dysfunction in our models of NASH because of leptin-induced miR21 expression (Fig. 4).

At this point, the results obtained reflected only a direct correlation of leptin, levels of miR21 and sinusoidal endothelial dysfunction. It was necessary that we definitively link the Leptin-miR21 axis in causing the dysfunctional endothelial in NASH models. To prove conclusively the role of leptin-induced miR21 as a key player in inducing sinusoidal endothelial injury and inflammation, we chose to use mice that were deficient in miR21 (miR21 KO) in the study. Also, we avoided in vitro knockdown of miR21 in a cellular model since we wanted to study a direct link of leptin induced miR21 in NASH disease pathology which would have been difficult to interpret in an otherwise in vitro cellular system. miR21 knockout mice showed significant upregulation of NOS3 phosphorylation and higher NOS3-p/NOS3 ratio in both the toxin model and dietary model of NASH (Fig. 5). Sinusoidal endothelial dysfunction markers VEGFR-2, E-selectin and ICAM-1 as shown by Pasarin R et al, were significantly decreased in miR21 knockout mice suggesting a direct role of miR21 in these events that are crucial for NASH progression.

NASH pathophysiology is a complex manifestation of multiple factors involved in regulation of sinusoidal endothelial function, inflammation, metabolic dysregulation and fibrosis [1,20]. Though our study shows conclusively that leptin-induced miR21 is involved in the development of sinusoidal injury in NASH, it falls short of explaining the exact mechanism of leptin in inducing miR21 in the liver. Though we have identified stellate cells as the primary cell type for mir21 upregulation in the liver, further studies in the whole animal using pharmacological approaches such as acetylcholine induced vasodilation in the liver sinusoids and primary cells in the liver are required to show leptin based mechanisms for induction of miR21. These may require identification of leptin or leptin-induced molecular mediators and their probable binding sites in the miR21 promoter sites.

Taken together, our study identifies miR21 as a regulator of sinusoidal endothelial injury, an early event in NASH pathophysiology in rodent models of NASH. The study also links higher hepatic leptin in inducing miR21 linked sinusoidal endothelial injury and inflammation, molecular events that are crucial for NASH development. The study can help advance the field of NASH pathogenesis by opening new avenues for research in the use of miR21 inhibitors as potential therapeutic agents.

## Supporting Information

S1 Fig *A*. 5×105 cells (primary hepatic stellate cells, LSECs and Kupffer cells) were incubated with leptin, LPS and Leptin+LPS. After 24 h incubation, lyzed cells were analyzed for miR21 expression using quantitative real time PCR. Data normalized against with only cells+medium control. *P<0.05 was considered statistically significant. *B*. Western blot analysis of cell lysates of hepatic stellate, LSECs and Kupffer cells incubated with leptin and LPS for mir21 target GrHL3. Data normalized against beta-actin immunoreactivity.
(PDF)

S2 Fig Cell lysates from liver stellate, LSECs and Kupffer cells were analyzed for sinusoidal injury markers. *A*. Red and Green columns show rat sinusoidal endothelial cell control and treated groups respectively. *B*. Yellow and light green columns represent stellate cells control and treated groups respectively. *C*. Blue and Orange columns represent rat Kupffer cells control and treated groups respectively. *D, E*, and *F* represent the CD34, VCAM1 and VEGF-a expressions respectively. Representative plot from 3 independent experiments.
(PDF)

S3 Fig Liver homogenates from DIO, DIO+BDCM and miR21 KO (treated with BDCM) at 1 weeks post BDCM administration groups were subjected to SDS page and western blot analysis. Representative blot from 3 experiments (n = 3).
(PDF)
