# Supplementary figures and images for: Upregulation of miR21 and Repression of Grhl3 by Leptin Mediates Sinusoidal Endothelial Injury in Experimental Nonalcoholic Steatohepatitis
Source: PLoS One. 2015 Feb 6;10(2):e0116780. doi: 10.1371/journal.pone.0116780 (PMC4319738; doi:10.1371/journal.pone.0116780)

Supplementary Fig. 1.

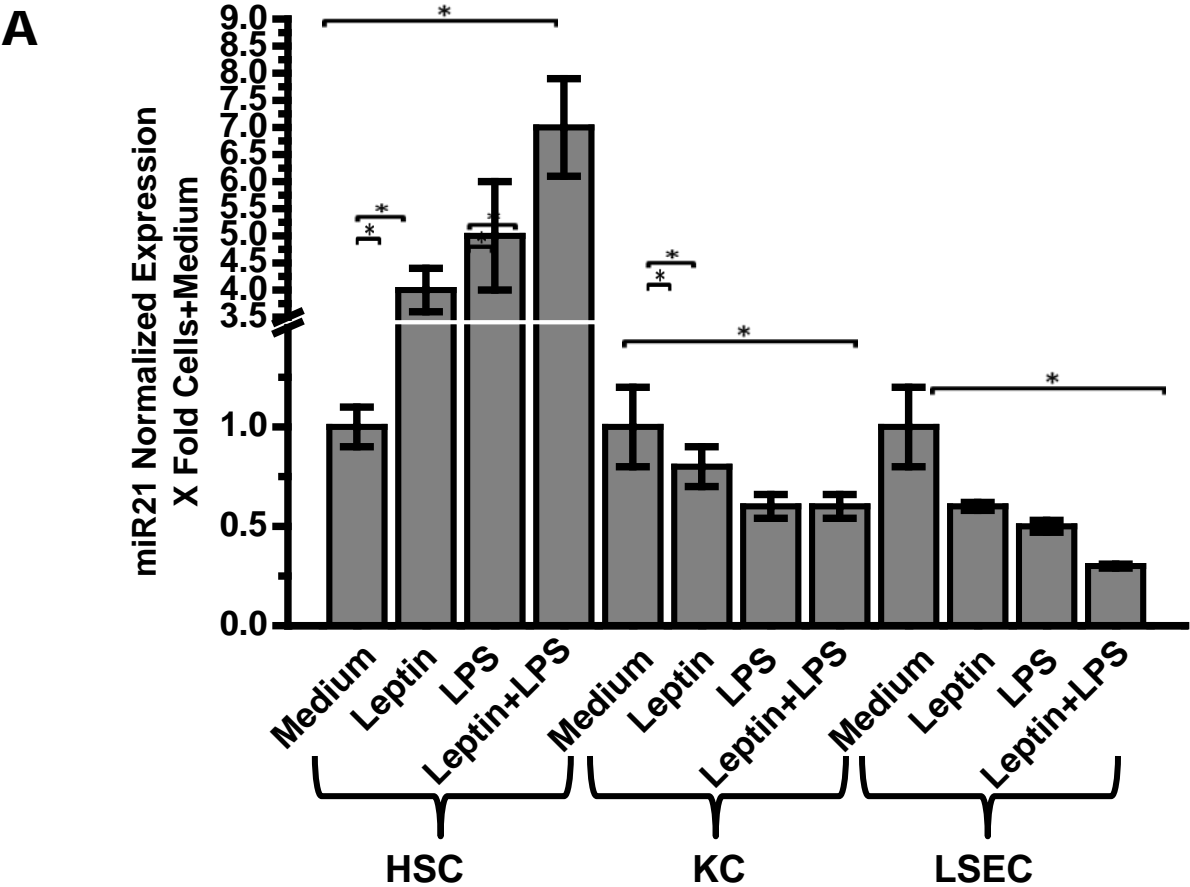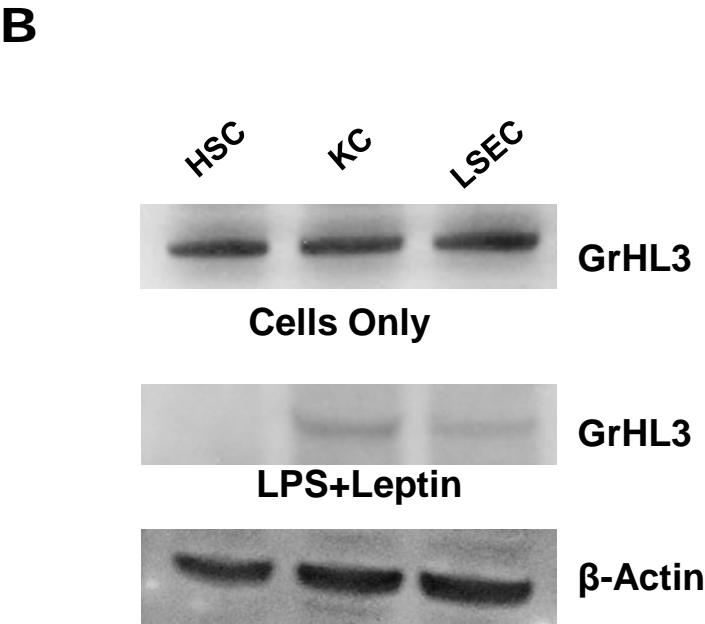

Supplement: S1 Fig — After 24 h incubation, lyzed cells were analyzed for miR21 expression using quantitative real time PCR. Data normalized against with only cells+medium control. *P<0.05 was considered statistically significant. B. Western blot analysis of cell lysates of hepatic stellate, LSECs and Kupffer cells incubated with leptin and LPS for mir21 target GrHL3. Data normalized against beta-actin immunoreactivity. (PDF) [file pone.0116780.s001.pdf]

Supplementary Fig. 3

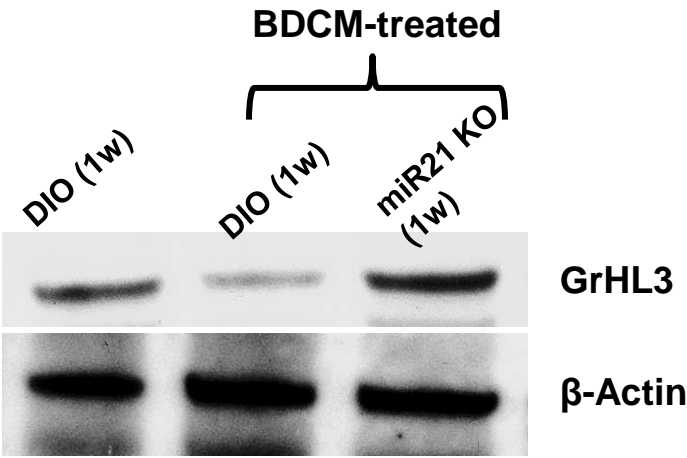

Supplement: S3 Fig — Representative blot from 3 experiments (n = 3). (PDF) [file pone.0116780.s003.pdf]
