# Supplementary material for: Upregulation of miR21 and Repression of Grhl3 by Leptin Mediates Sinusoidal Endothelial Injury in Experimental Nonalcoholic Steatohepatitis
Source: PLoS One. 2015 Feb 6;10(2):e0116780. doi: 10.1371/journal.pone.0116780 (PMC4319738; doi:10.1371/journal.pone.0116780)
Supplement: S2 Fig — A. Red and Green columns show rat sinusoidal endothelial cell control and treated groups respectively. B. Yellow and light green columns represent stellate cells control and treated groups respectively. C. Blue and Orange columns represent rat Kupffer cells control and treated groups respectively. D, E, and F represent the CD34, VCAM1 and VEGF-a expressions respectively. Representative plot from 3 independent experiments. (PDF) [file pone.0116780.s002.pdf]

Supplementary Fig. 2

A

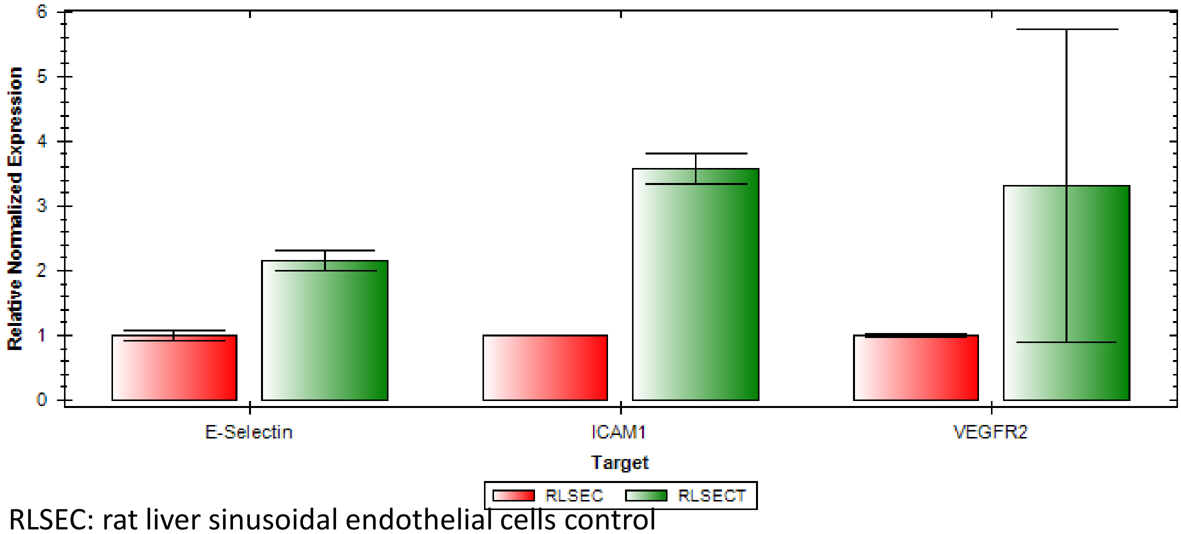

RLSEC: rat liver sinusoidal endothelial cells control

RLSECT: rat liver sinusoidal endothelial cells treated (lps+leptin)

B

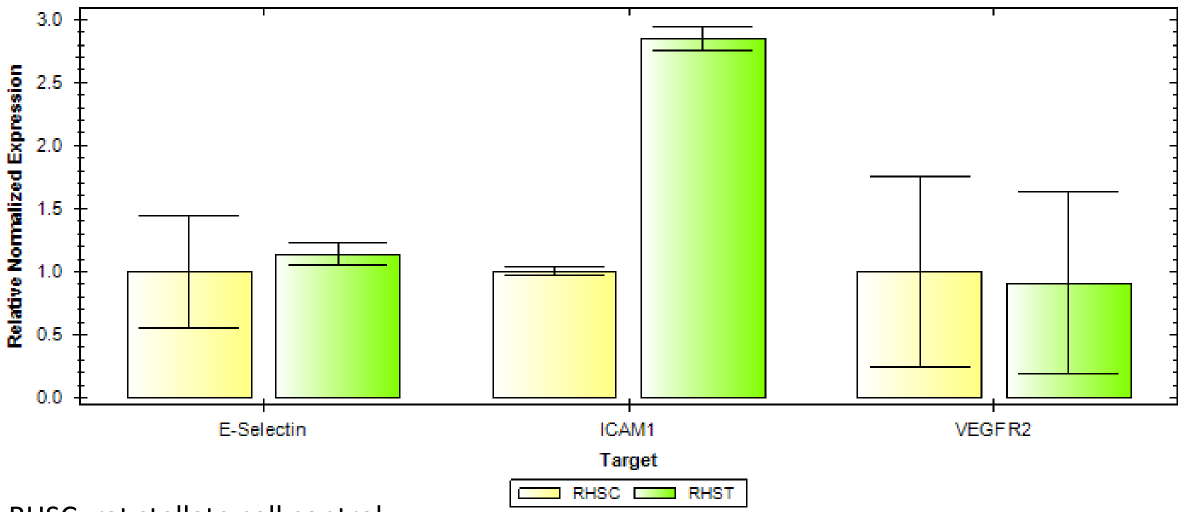

RHSC: rat stellate cell control

RHST: rat stellate cell treated (lps+leptin)

C

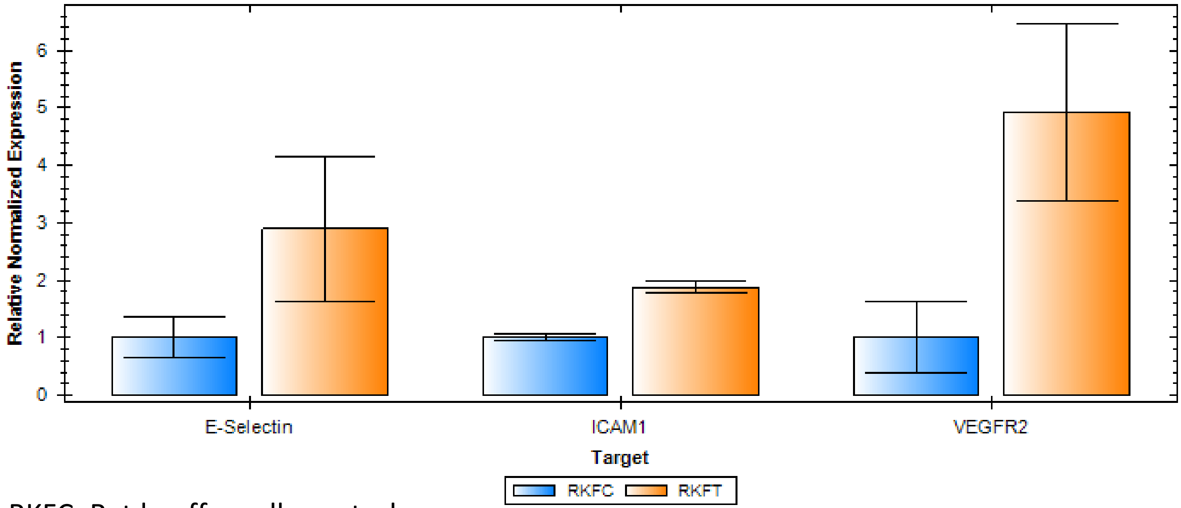

RKFC: Rat kupffer cells control

RKFT: rat kupffer cell treated (lps+leptin)

D

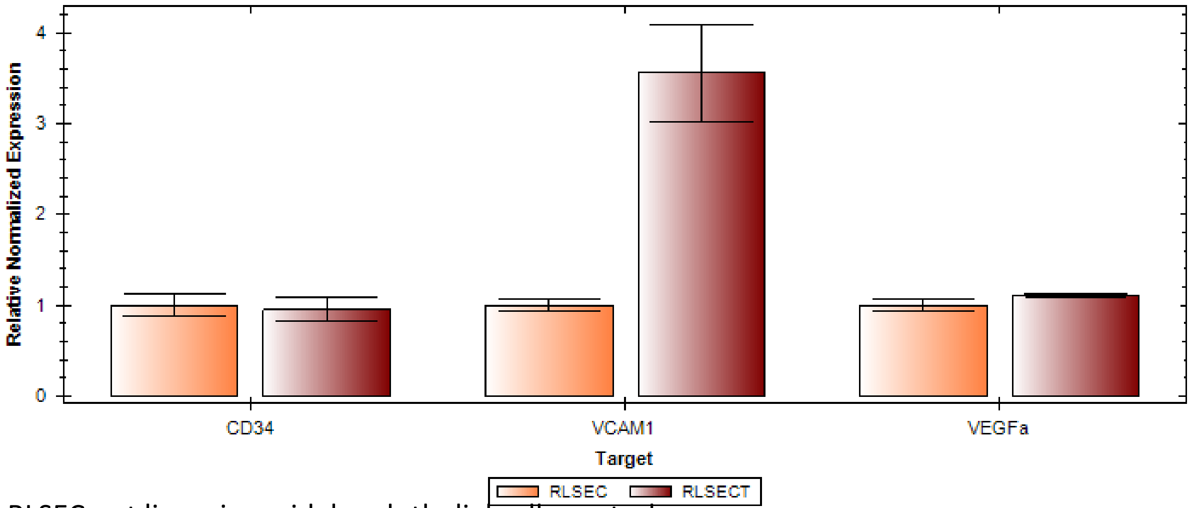

RLSEC: rat liver sinusoidal endothelial cells control

RLSECT: rat liver sinusoidal endothelial cells treated (lps+leptin)

E

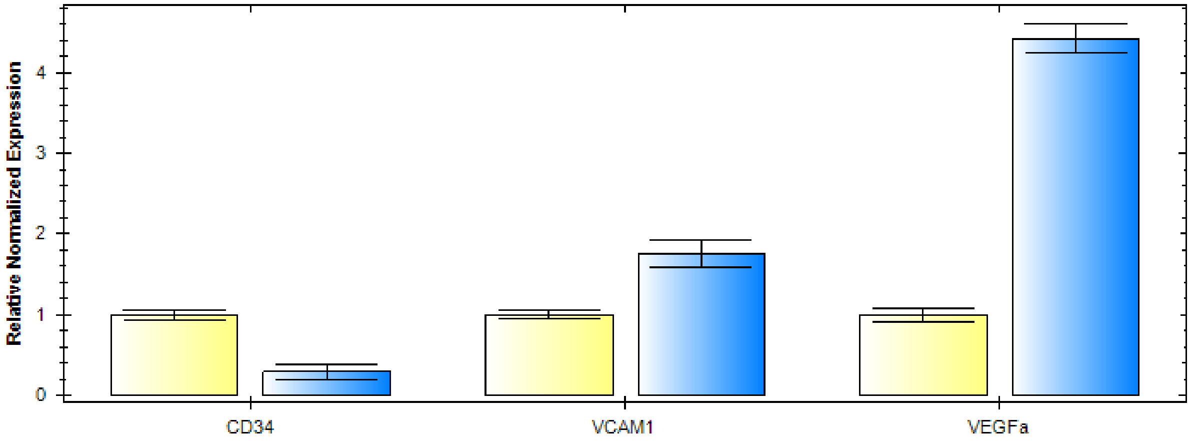

RKFC: Rat kupffer cells control

RKFT: rat kupffer cell treated (lps+leptin)

F

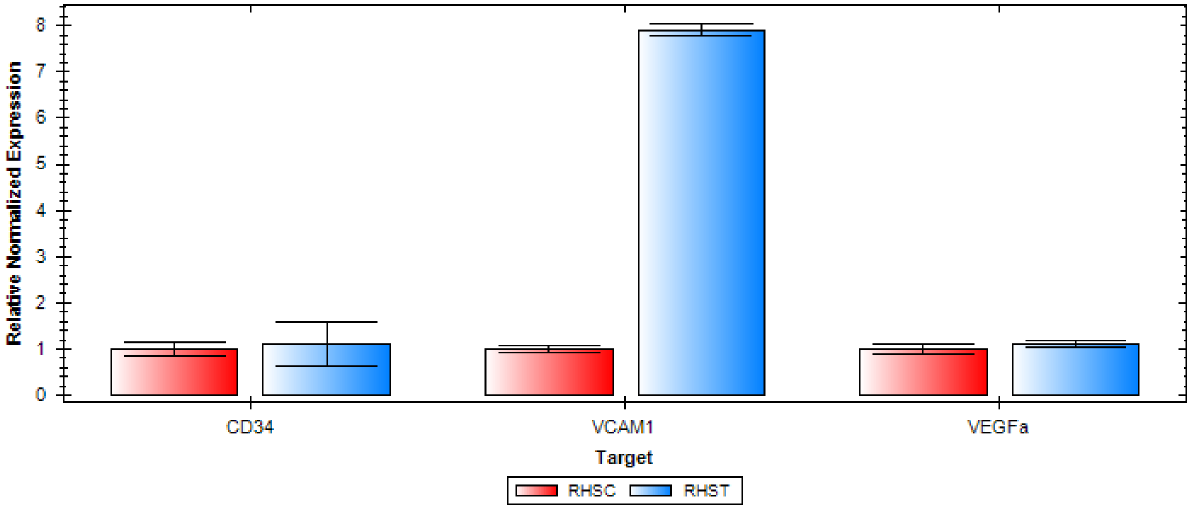

RHSC: rat stellate cell control

RHST: rat stellate cell treated (lps+leptin)
